# COVID-19 Era Social Isolation among Older Adults

**DOI:** 10.3390/geriatrics6020052

**Published:** 2021-05-18

**Authors:** Stephanie MacLeod, Rifky Tkatch, Sandra Kraemer, Annette Fellows, Michael McGinn, James Schaeffer, Charlotte S. Yeh

**Affiliations:** 1 Research for Aging Populations, Optum Labs, Ann Arbor, MI 48108, USA; rifky.tkatch@optum.com (R.T.); michael.mcginn@optum.com (M.M.); jamie.schaeffer@optum.com (J.S.); 2 United Healthcare Medicare & Retirement, Minneapolis, MN 55440, USA; sandra_kraemer@uhc.com (S.K.); annette_fellows@uhc.com (A.F.); 3 AARP Services, Inc., Washington, DC 20049, USA; cyeh@aarp.org

**Keywords:** older adults, pandemic, COVID-19, social isolation, social connectedness, social recession

## Abstract

Risk of COVID-19 exposure and more severe illness are serious concerns for older adults. Social distancing has worsened existing social isolation, with severe impacts on connectedness among seniors. The pandemic is threatening to cause an extended health crisis, with impacts including serious health consequences. Our primary purpose is to summarize emerging research describing the impacts of the pandemic on social isolation among older adults. A streamlined search was conducted to fit the scope of this literature review. Common research databases and mainstream resources and websites were utilized to identify research published or released in 2020 to align with the pandemic. Early research indicates that the pandemic has worsened social isolation among older adults. Social isolation has become urgent, as seniors have lost their usual connections due to social distancing. While safety measures are critical to prevent virus exposure, this approach must be balanced with maintaining social connectedness. The pandemic highlights the importance of social connections, with significant impacts on both community-living older adults and those in nursing facilities. Safety protocols have created a paradox of reduced risk along with greater harm. Consequently, adapted approaches are urgently needed to address the consequences of a long-term social recession.

## 1. Introduction

The need for social connectedness among older adults has been studied extensively; experts state that being socially connected is critical to health and survival [1,2,3]. Social connectedness plays a prominent role in preventing both loneliness and social isolation, with social isolation specifically defined as an objective measure of one’s social interactions, relationships, networks, and support [2,3]. Social isolation is estimated to impact up to 40% of adults aged 60 and older in the US [2]. According to the National Health and Aging Trends Study report, released in early 2020 just before the novel coronavirus (COVID-19) outbreak, 28% of older adults aged 65+ in the US (9 million) were already socially isolated at that time [4,5,6].

The negative impacts of social isolation later in life have been widely reported, ranging from declining mental and physical health to reduced quality of life, increased mortality, and higher Medicare costs [7,8,9,10]. Older adults who live alone, with limited resources and cognition/memory issues, are at even greater risk of social isolation, worsened significantly by the COVID-19 pandemic [11].

Worldwide spread of the pandemic triggered an urgent, ongoing public health crisis in the US and worldwide, beginning in early 2020 [12]. Significant effects include millions of infections along with widespread, serious illness and death; devastating strain and shortages within the healthcare system; severe economic ramifications; growing mental health concerns; and strict social distancing efforts leading to greater social isolation among the most vulnerable individuals [12].

Older adults have been disproportionately impacted by COVID-19, with higher susceptibility to severe illness, hospitalization, and death [13]. In the US, those who are age 65+ comprised about 80% of all COVID-19 deaths as of late 2020; worldwide, estimates suggest that 95% of pandemic related deaths have been among those age 60 and older [13,14]. While the physical illness and death impact has been emphasized, the pandemic has taken a toll on mental health and quality of life, as well [3,12,15].

Social distancing has significantly impacted social connectedness especially among seniors who are already isolated [3,15]. Although recommendations have varied by state and by urban versus rural areas, social distancing has been especially important for vulnerable older adults due to their high risk of severe illness resulting from COVID-19 infection. Thus, from early in the pandemic, older adults in most areas have been urged to stay home as much as possible until recent availability has improved access to approved COVID-19 vaccines. Consequently, increased social isolation has added to family and caregiver burden, reduced access to healthcare services, strained support networks, and hampered coping strategies for many [16]. Older individuals who previously left their homes regularly for social activities, volunteering, doctors’ appointments, family visits, and errands have generally suspended these activities due to strong recommendations and strict guidelines urging older Americans to stay home. Thus, their social connectedness, one of several factors recently identified to be key personal determinants of health [17], has been severely impacted. Notably, a collection of personal factors has been defined as personal determinants of health, or PDOH—an emerging concept built on the individual resources that help buffer challenges in life and support optimal health outcomes [17]. In addition to social connectedness, other important PDOH including resilience and purpose in life may potentially decrease over the long term for many individuals as a result of the pandemic, with the abrupt absence of normal support systems, roles, and activities [3,15,17]. Furthermore, older adults who are hospitalized for COVID-19 infections with a poor prognosis may not be able to perform life completion and end-of-life tasks due to current hospital restrictions such as prohibited family visits, creating a reality that may reduce quality of life for the patient and cause distress for family members [16].

The pandemic has made these realities increasingly challenging, as every area of life has changed in efforts to slow the spread of the virus [3]. Downstream consequences include not only an economic recession, but also the potential for a social recession, recently defined as a lasting collapse in social contact resulting from compounded social isolation, especially among the most vulnerable [3,12,18]. A long social recession could worsen the well-known negative impacts of social isolation, encompassing both physical and mental health outcomes as well as longevity and overall quality of life [3,12,18].

UnitedHealthcare (UHC) and AARP Services, Inc. (ASI) work together to help improve health outcomes, quality of life, and reduce costs and utilization for a growing population of older Americans. To that end, a key goal is to address the unique needs and health concerns of older adults covered under AARP^®^ Medicare Supplement Plans insured by UnitedHealthcare Insurance Company or an affiliate (collectively “UnitedHealthcare”). Thus, considering the ramifications of the COVID-19 pandemic crisis in the US, this literature review examines related effects on social connectedness among older adults.

## 2. Statement of Purpose

Our primary purpose is to describe the immediate and lasting impacts of increased social isolation among older adults resulting from the COVID-19 pandemic, social distancing, and the subsequent loss of social connectedness and relationships as well as negative effects on physical and mental health. This review will also briefly explore potential approaches that could be adapted for safety protocols to combat social isolation among older adults in this unique time, as well as the need to consider the long-term impacts of the pandemic and its lasting consequences on health outcomes. In doing so, we aim to present opportunities to address an emerging social recession with approaches tailored for use during the pandemic and beyond.

We hypothesize that: (1) the COVID-19 pandemic has compounded and worsened social isolation among older adults, in large part due to their susceptibility to severe illness and death as a result of infection with the virus as well as existing social isolation challenges and orders to stay at home; and (2) the impacts of intensifying social isolation within this population have the potential to be long-lasting, with subsequent consequences for mental and physical health outcomes.

## 3. Methods

For this review, a targeted literature search was conducted to focus on the specific topics of interest, rather than a broad systematic review of all related areas (such as all social isolation). Per PRISMA 2020 guidelines, instead of a systematic review, this was conducted as a scoping review of literature to provide an overview of new, emerging literature on our topic areas of interest—a methodology utilized due to the recent and limited nature of published literature specific to the COVID-19 pandemic. However, the following guidelines for qualitative literature review studies were considered:

- Provide a statement of the objective or hypothesis the review addresses.
- Specify the inclusion and exclusion criteria for the review and how studies were categorized by search terms.
- Identify the databases, websites, organizations, and other references searched or utilized to select relevant studies.
- Specify methods used to decide whether a study met the inclusion criteria for the review.
- Describe the search and selection process, using a flow diagram to include the number of studies ultimately included as citations in the review.
- Cite each study included along with full reference information.

Other guidelines for methodology of research studies were not applicable if they pertained to data collection, surveys, data analysis, or other processes, as our literature review did not integrate those steps.

Notably, older adults in the US were our target population; thus, research focused on other age segments and locations was excluded. Our search gathered articles published from early 2020 through year-end 2020 in order to align with the onset of the COVID-19 pandemic.

Established research databases and search engines were utilized, primarily PubMed, Google, and Google Scholar, as well as updated mainstream websites as appropriate to identify recently released content. Included were legitimate websites maintained by the World Health Organization (WHO), Centers for Disease Control and Prevention (CDC), AARP and AARP Foundation, Commonwealth Fund, Gerontological Society of America (GSA), and other reputable organizations. Search tools were selected for the scope of publications available, timeliness of research, access to articles, and alignment with standard literature review methodology.

Search terms and phrases were identified and streamlined to identify publications that closely aligned with our focus. An initial list of terms was determined by considering key areas of interest, specifically focusing on social isolation and connectedness among US older adults during the COVID-19 pandemic.

**Figure 1:**
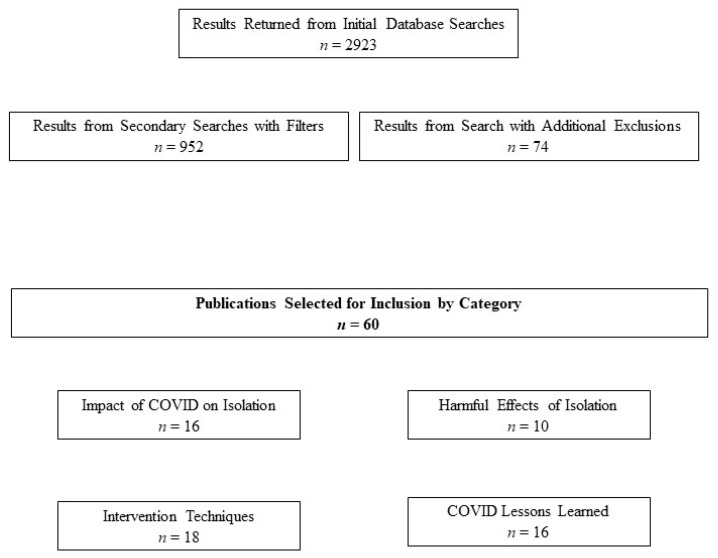
Diagram Displaying Search Methods and Results.

Several search terms returned numerous results, many of which were outside the scope of this paper. However, these initial results provided an overview of the emerging body of research on the impacts of the pandemic on social isolation among older populations. Subsequently, we further narrowed these initial results to identify publications most closely related to our purpose including those focusing on impacts of the COVID-19 pandemic on social isolation during 2020. We used PubMed’s advanced search feature with the Medical Subject Headings (“MeSH”) filter to further narrow results. Next, we further streamlined results with the MeSH Major Topic option to identify more relevant publications for several topics. Titles and selected abstracts were reviewed as needed; selected reference lists of the most relevant articles were also reviewed for potentially useful resources. Inclusion criteria included original research and review publications with titles and/or abstracts that fit our scope, as well as those focusing on older populations (i.e., primarily age 60+ but also age 50–60). Research focusing strictly on younger populations or other specific demographic groups was typically excluded. Exclusions also encompassed articles published outside the US, general research on topics unrelated to the pandemic except for background content, as well as those published prior to 2020. Additionally, only articles published in English were selected. Table 1 displays examples of the search terms used; the number of filtered results identified for each term or phrase are also listed. Meanwhile, Figure 1 displays the search methods and results obtained in a pictorial format.

## 4. Summary of Results

### 4.1. The Shift in Social Connectedness during COVID-19

Emerging literature identified in this review confirms our previous hypotheses that: (1) the COVID-19 pandemic has compounded and worsened social isolation among older adults; and (2) the impacts of intensifying social isolation within this population have the potential to be long-lasting, with subsequent consequences for both mental and physical health outcomes. Research conducted during the early months of the pandemic and its accompanying stay-at-home orders and lockdown conditions demonstrate these impacts, with growing consensus on the key concerns escalating during this unprecedented era.

**Figure 2:**
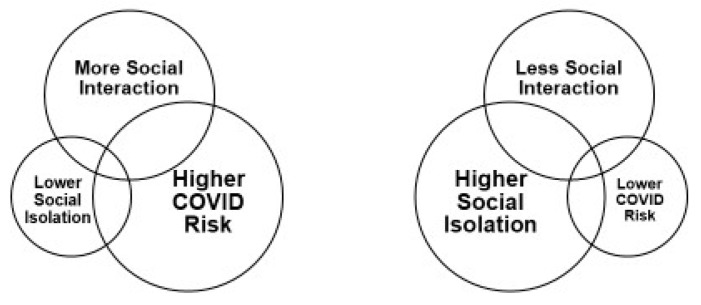
The COVID-19 Social Connectivity Paradox^20^.

Social isolation is a well-known concern within older populations, yet its impacts on physical, psychological, and social health gained greater awareness at the onset of the COVID-19 outbreak [19]. Since early 2020, the pandemic has truly highlighted the importance of social connections for seniors. Social isolation quickly became even more urgent as older adults lost their usual connections due to quarantine orders and social distancing recommendations designed to keep them safe. As such, a new phenomenon has occurred across this age group, as the safety protocols executed to protect older adults have subsequently placed them at greater risk of social isolation. The new concept emerging to describe this cycle resulting from social distancing efforts has become known as the COVID-19 Social Connectivity Paradox [20] (Figure 2).

Within this paradox, frequent interaction with others prevents social isolation, but also leads to higher risk of COVID-19 infection. Conversely, reduced interaction (due to social distancing and staying home) leads to greater isolation, yet lower risk of exposure [20]. Compounding these factors, family members and friends of older adults often stay away and stop in-person visits to avoid exposing aging loved ones to the virus [15,20]. The consequences of this paradox require urgent attention as it intensifies the existing problems of social isolation and disconnectedness among seniors; solutions adapted for safety are needed now and in the longer term [20].

Another new phenomenon resulting from the pandemic has been described as “Lockdown Loneliness,” referring to loneliness specifically caused by worsening social isolation and disconnectedness due to lockdowns and stay-at-home orders [21]. This trend is supported by June 2020 data from Great Britain suggesting that “Lockdown Loneliness” impacted 7.4 million residents at that time [21]. Chronic loneliness remained at pre-lockdown levels (2.6 million), but among these lonely individuals, 80% were further impacted by pandemic lockdowns [21].

At the same time, experts predict a long-term societal impact, compared to an economic low period and referred to as a “social recession,” or the long-term collapse in social contact and fraying of social bonds that could lead to worsening chronic isolation as the loss of interaction continues over time [3,12,18]. A social recession could have lasting impacts on both physical and mental health, with serious consequences expected among the oldest and those in poor health. Unfortunately, the effects of a social recession are likely to be harder to assess and reverse than those of an economic recession.

Meanwhile, new research emphasizes the unique challenge of social isolation in long-term care settings, compounded by safety guidelines since the start of the COVID-19 outbreak. During the pandemic, infection rates and deaths among long-term care residents were initially overwhelming, with early “hot spots” of the virus in many of these facilities [22,23,24]. Estimates from the Centers for Disease Control and Prevention (CDC) indicate that long-term care residents comprised over 25% of all COVID-19 related deaths in the US as of late 2020 [25], while in Europe and Quebec, Canada, rates appear even higher: 50% and 88%, respectively, of COVID-19 deaths have occurred in these facilities [26,27]. Consequently, restrictions to prevent additional spread of the virus typically prohibit visits from family members and friends [22]. At the same time, residents are often confined to their rooms inside facilities, with a sudden halt in group activities and gatherings for meals to reduce infection rates [22]. Researchers predict that these factors will lead to significant consequences for older adults, worsening not only social isolation but also levels of loneliness, stress, anxiety, and depression, potentially further compounding physical health conditions [22,28,29].

### 4.2. Assessing Social Isolation and the Impacts of COVID-19

As the pandemic worsened throughout 2020, fast-track research aimed to quickly examine the widespread impacts of COVID-19. Most research published prior to the end of 2020 primarily describes small survey studies and commentaries yet provides a foundation for growing knowledge [30,31,32]. As one focus area, researchers emphasized the need to quickly assess social isolation among vulnerable seniors amid quarantines and social distancing recommendations [30,31,32]. Experts stress that both during the pandemic and as normalcy returns, providers must regularly screen for social isolation and take proactive steps to alleviate it [19]. In this effort, several surveys tailored with COVID-related questions have been developed in small studies. One such measure is the Questionnaire for Assessing the Impact of the COVID-19 Pandemic on Older Adults (QAICPOA), a 17-item survey [30]. The QAICPOA was designed for use on the telephone with older participants to assess the impact of social isolation during COVID-19 and is currently freely available to use in subsequent studies. The 17 items focus on changes in social support during the pandemic, encompassing both emotional (who provides it) and instrumental (such as how food and medication is obtained while quarantined) [30]. The QAICPOA is currently being applied in part to assess needs and barriers to connectedness, as well as to ensure referrals to resources.

Another new measure, the Fear of COVID-19 Scale (FCV-19S), is a seven-item survey designed to assess emotional, cognitive, and behavioral impacts of the pandemic. Developed for the general population, the FCV-19S uses a five-point scale with responses from strongly agree to strongly disagree, with higher scores indicating greater fear about the virus [33]. Questions include: “I am most afraid of coronavirus-19;” “It makes me uncomfortable to think about coronavirus-19;” “My hands become clammy when I think about coronavirus-19;” and others.

Finally, the Understanding America Study (UAS) COVID-19 Survey was conducted in April–May 2020 among US adults age 50+ (*n* = 3283) [34]. This survey, funded by the National Institutes of Health (NIH), evaluated the impacts of social distancing measures primarily on loneliness. One single item asked, “In the past 7 days, how often have you felt lonely?” with responses of at least one day coded as lonely. Those who stated they were implementing social distancing measures, including cancellation of social activities and avoiding contact with others, reported up to 41% greater loneliness.

### 4.3. Growing Impacts of the Pandemic on Older Adults

Meanwhile, several small research studies have attempted to determine how the pandemic is affecting older adults’ lives, including levels of social isolation. In one study, researchers conducted an analysis of online focus group discussion data from older adults with pre-frailty or frailty while under stay-at-home orders in March 2020 (*n* = 10; total posts = 60). Topics in the online posts included impacts of the pandemic on daily life, preparedness, and technology use [11]. Participants reported increased stress, anxiety, and lack of preparedness for the crisis, along with greater feelings of social isolation due to ongoing restrictions. However, many were increasingly using technology for social connections and to find current information [11]. Another study evaluated the impact of early COVID-19 restrictions on older adults with pre-existing depression. Telephone interviews (*n* = 73) were conducted with individuals who had previously taken a survey before the pandemic related to their depression. In this follow-up survey, most participants described increasing depression, stress, anxiety, and lower quality of life (QOL), despite feeling somewhat resilient or positive at first, which authors suggested could indicate a “honeymoon” phase of resilience [35]. Nevertheless, most also reported concerns and anxiety about the future of the pandemic and ongoing spread of COVID-19, along with continued restrictions to their routines [35].

Similarly, an early 2020 study evaluated the short-term effects of sheltering in place [36]. Older adults (*n* = 93) had previously completed surveys on loneliness and social networks prior to the start of the pandemic as part of a mental well-being study. Then, in April and May 2020, study participants completed the same items by phone, as well as new items related to COVID-19. Most participants reported increased depression and greater loneliness following the onset of the pandemic, as compared to 6–9 months prior. In addition, loneliness positively predicted increased depression during stay-at-home orders [36]. 

Another online survey evaluated perceptions of threats related to the pandemic among over 1700 adults in the US, of all ages (18–89 years) [37]. Overall, perceptions of COVID-19 as a threat to health were high across all age groups. Notably, the greatest perceived negative impacts of the pandemic were among the oldest and associated with stress, loneliness, and poor sleep. Meanwhile, a survey conducted in July examined other potential effects of the pandemic, specifically disruptions in healthcare services among those age 65 + due to concerns about COVID-19 exposure [38]. Participants reported disruptions to care including mental health care (69%), dental care (67%), primary care (63%), and rehabilitation (63%). Of those reporting disruptions to care during the pandemic, 35% reported the reason as fear of contracting the virus and becoming seriously ill. Over half of survey respondents were using telehealth options; of those, 95% reported satisfaction with the technology used [38], suggesting that telehealth expansion could increase access to routine care now and in the future.

Meanwhile, perspectives are also emerging on the potential for long-term effects of the pandemic on both mental and physical health, resulting from daily lifestyle changes [39]. For instance, reduced exercise while sheltering in place could lead to increased weakness, reduced mobility/balance, and thus higher falls risk [39]. Shifting availability of healthy, fresh food options with older adults staying home instead of shopping frequently could have a potential impact on heart health, diabetes, and weight. In addition, the reduction in support services, caregiving, and opportunities for socialization will likely worsen loneliness, social isolation, and depression over time; fear of virus exposure has already amounted to missed preventive care appointments and procedures [39]. Finally, authors of a recent commentary suggest that the pandemic and post-pandemic environment may present a combination of factors known to increase suicidal behaviors or ideation among isolated seniors [40]. These factors include living alone, loneliness, and disconnectedness from society; loss of social opportunities; and reduced access to mental health resources [40].

### 4.4. Exploring Immediate Solutions during a Pandemic

Considering the consequences of social isolation worsened by the pandemic, it is important to consider immediate outreach options. An approach being leveraged at this time focuses on volunteer phone outreach to engage older adults who are isolated at home. For example, one study described the development of a brief single-session intervention to deliver a “Connections Plan” program to engage socially isolated seniors and help them cope with social isolation during COVID-19 [32]. This study used the QAICPOA assessment along with cognitive behavioral therapy (CBT) strategies to design the intervention [30,32]. In this approach, research clinicians work with participants remotely using strategies to cope with social isolation, by teaching various tools by phone, email, or mailed handouts. Skill building focuses on changing the individual’s perspective, actions, and body sensations to cope with feelings of isolation. Although under development at the time of this review, this approach has the potential to provide social connectedness and coping tools for seniors [32].

Other new studies have quickly implemented telephone outreach programs to help socially isolated seniors since early 2020. One program recruited medical and healthcare student volunteers to call older adults at risk of isolation (*n* = 25) during the COVID-19 crisis [41]. The program, Seniors Overcoming Social Isolation (SOS), identified at-risk seniors through their healthcare providers, targeting both community-dwelling adults and those in long-term care facilities. Student volunteers used a script with conversation starter topics including personal history, health issues, technology use, family/friends, and COVID-19 concerns. In early results, older adult participants expressed appreciation for the calls/callers; student volunteers felt the calls were well received and also reported personal satisfaction [41].

Another similar program was designed to address social isolation specifically among nursing home residents during the COVID-19 outbreak [42]. The Telephone Outreach in the COVID-19 Outbreak (TOCO) program was developed to provide weekly telephone calls by medical student volunteers, with the goal of alleviating social isolation in nursing homes worsened by quarantine of residents in their rooms [42]. Local nursing homes were contacted; at selected facilities (*n* = 3 facilities), recreation directors identified at-risk residents who were willing to receive weekly phone calls (*n* = 30 participants). Initial results were positive; participants reported that they looked forward to weekly phone calls with the student volunteers and expressed gratitude for the connections made with them. In fact, the student group in this study plans to continue the TOCO program beyond the pandemic to address social isolation in nursing homes [42].

Meanwhile, other technology-based options have targeted social isolation, both prior to and during the pandemic. One recent literature review (2020) summarized the 15 smartphone applications most highly recommended as effective for addressing seniors’ needs and providing access to needed support while isolated at home [43]. Many of these selected applications are highlighted in Table 2, although the availability of various application options continues to expand. Research indicates that seniors are increasingly using mobile technology, including smartphone applications, for healthcare purposes as well as to stay connected and access resources during this time [43,44]. Thus, it is reasonable to consider that mobile technology could be useful for those who want to avoid leaving home while staying connected and maintaining access to healthcare services, medications, groceries, and other resources.

Recent efforts are also exploring the benefits of online peer-to-peer communities for older adults, especially during this time of increased social isolation and disconnectedness. Online communities and social networks, part of the collective technological tools known as information and communication technologies (ICT), have become popular among older adults in recent years, especially since the start of the pandemic [45]. One recent review compiled studies evaluating the effectiveness of ICT with older adults, finding that ICT had a positive impact on social support, social connectedness, and social isolation over the short term [2]. Generally, online communities connecting older adults to their peers demonstrate success in sharing information, experiences regarding health conditions, and peer support for those with common interests [45]. One ongoing study, which began following the onset of COVID-19 in April 2020, is exploring the feasibility of online communities for older adults as a medium to connect peers with one another and communicate with healthcare providers [45]. Results describing whether these communities and social networks play a beneficial role in connectedness are expected in late 2021. 

Finally, innovative options to address social isolation during the pandemic and beyond have been proposed. As one novel example worth mentioning, “sociable robots” have been suggested as companions for seniors who are socially isolated, even prior to the COVID-19 outbreak. Since the pandemic began, life-like sociable robots have been discussed primarily as a solution for use in COVID-19 testing and diagnosis, telemedicine, cleaning, decontamination, and monitoring of quarantine. However, some perspectives recommend the use of robots as social companions for older adults confined to their homes. Essentially, robots could function as companions in an innovative approach to reach seniors who are lonely and isolated, as well as to provide simple task-focused functions or assistance. Although this approach would present various challenges, it appears in various discussions about future directions for alleviating social isolation [28,46,47,48]. Taken together, the commentaries and studies described here demonstrate the research quickly emerging since the start of the pandemic that could help inform solutions for supporting socially isolated older adults during a challenging time.

## 5. Discussion

The COVID-19 pandemic triggered a public health crisis worldwide, impacting every aspect of life, society, health care, and individual health outcomes. While older adults have always been vulnerable to social isolation, the pandemic era highlights an urgent need to boost social connectedness within this population. Notably, social isolation has long been confirmed as detrimental to overall health, especially among vulnerable seniors who already face multiple chronic conditions and declining health status that amount to greater susceptibility for isolation [3,5,15]. Furthermore, a growing body of literature confirms social isolation as a significant negative influence not only on mental health but also on physical health outcomes, with social isolation and loneliness associated with higher risk of dementia, coronary artery disease, stroke, and mortality [3,5,15]. Thus, the need to explore pandemic-era intensification of social isolation among already vulnerable older adults was recognized as an urgent need, forming the focus of this literature review.

Immediate impacts of worsening social isolation since the pandemic began have emerged: from mental health and emotional distress to problematic health behaviors including poor sleep [3]. Notably, the pandemic environment requires innovative strategies to be effective despite social distancing and quarantines. Even after the height of the pandemic has passed, seniors who are still afraid to leave home may struggle to re-establish their prior social connections, relationships, and usual activities.

### 5.1. Adapting Approaches for Safety

Most new research describing the impacts of COVID-19 on social isolation emphasizes that approaches and solutions must be adapted for safety due to the pandemic. Furthermore, immediate solutions are needed to address the potential for a lasting social recession, integrating efficient and accurate identification of those who are socially isolated and in greatest need of support. Traditional approaches may not be feasible when social distancing efforts are in place; in fact, in-person interactions may be limited for the foreseeable future. In addition, emerging studies agree that adapted approaches for the pandemic should be multidimensional, with planning for longer-term delivery; innovative strategies for safe, effective outreach; and the use of public health messaging and virtual communication to ensure continued contact with friends, family, and community resources [11,12,35,49,50]. Thus, a growing need exists for what is termed “distanced connectivity” in the literature. Distanced connectivity as an approach aims to repair or improve aspects of social connectedness damaged by social distancing through technological resources. At a scalable level, technology-based options may be a logical approach to engage older adults in efforts to reduce feelings of isolation. Furthermore, initiatives using technology can help to address older adults’ needs during the current pandemic and beyond, both individually as well as in healthcare delivery on a larger scale [51].

Technology may also provide desperately needed resources for those in long-term care settings. In one recent study, 100% of a nursing home’s activity staff members reported that their residents were impacted by social distancing inside the facility during the pandemic [52]. Staff members expressed eagerness to use any feasible technology-based solutions to connect with residents, including digital therapy sessions; telehealth provider visits; virtual/digital announcements; video calls between residents and to outside friends/families; and virtual activities across residents’ rooms [52]. Elsewhere, related research suggests that technology options for enabling communication in nursing homes may become part of the new normal following the pandemic, with video consultations and visits remaining important for both family and provider connections [22,53,54].

Similarly, home health and medical home visit programs have had to rely on technology, in many cases, during the pandemic. As an example, the Division of Geriatrics at Staten Island University Hospital typically provides regular, recurring home visits for over 300 families in the area [55]. Early in the pandemic, the program faced numerous barriers including fear among patients and families of accessing the healthcare system through home visits due to potential COVID-19 exposure, as well as decreased diagnostic support and reduced ability to deliver home visits [55]. However, technology has served an important role in executing these services through telehealth, which most families have embraced successfully, according to physicians employed by the program [55].

Finally, the value of technology in palliative care during this time has been described, focused on the need for delivering continued quality while also maintaining safety protocols [16]. For older adults with severe COVID-19 illness and poor prognosis, end-of-life tasks and family communications have relied on technology in the clinical setting. For example, one case study described a hospitalized patient who subsequently died from COVID-19 complications, and the role of video conferencing for consultations as well as provider and family visits while he was ill [16]. Guided imagery, meditation, and prayer were also delivered to this patient through virtual platforms in the hospital, which reportedly reduced his anxiety and distress at the end of life [16].

Meanwhile, in other efforts, the Administration on Aging (AOA) and the National Council on Aging (NCOA) provided recommendations in early 2020 for aging organizations to adapt their resources during the COVID-19 pandemic [20,56,57]. The AOA and NCOA released a coordinated series of toolkits, webinars, and other resources to help organizations pivot by offering virtually delivered services instead of in-person outreach. As an example, the NCOA’s Program to Encourage Active, Rewarding Lives (PEARLs) was converted to a virtual format in March 2020 to adapt to the rapidly evolving COVID-19 situation. Designed for older adults, the PEARLs program addresses late-life depression in order to prevent worsening social isolation, through teaching problem-solving and activity planning skills virtually, helping seniors create a “new normal” without in-person interactions.

With similar goals, AARP’s Community Connections website has increasingly focused on connecting older adults with their communities during a time of greater social isolation [58]. Older individuals seeking support for various needs can find it through this website; those interested in helping can also reach out and connect with their communities. One program linked through this website is AARP’s Friendly Voice, a phone line that can connect seniors impacted by social isolation resulting from social distancing and quarantines during the pandemic. In this program, trained volunteers are available to make calls to anyone who requests contact. Those in crisis or with urgent needs (such as food, health care, and mental health support) are directed to appropriate experts or resources by volunteers. However, most calls are intended to provide a “friendly voice,” or just someone to talk to during a time of extreme stress and growing isolation [58].

Despite the potential benefits of interventions tailored to avoid in-person contact, various challenges exist with some of these options. Notably, unreadiness among older adults to access telehealth is common for various reasons: privacy or security concerns; access to internet-enabled devices; lack of knowledge for using devices and/or platforms; and hearing/vision impairments [59]. The costs of telehealth along with implementation logistics may also pose unique challenges; however, telehealth visits may help improve access to healthcare services and remote monitoring even beyond the pandemic [11,12,35,49,50,51].

Elsewhere, recent discussions have considered how healthcare providers can help address the ongoing impacts of social distancing, quarantines, and COVID-19 fears through various approaches [20,39]. First, providers can improve telehealth visits by reminding patients to wear glasses and hearing aids; engaging caregivers in three-way calling; and enlisting help with video technology from family members [20,39]. Alternatively, in-person visits and home health evaluations can continue safely as long as safety regulations remain in place. Finally, providers can help ensure access to necessary resources by asking about social determinants and providing needed connections [20,39].

Finally, with the availability of COVID-19 vaccines in the US, it will be critical to reach socially isolated older adults to ensure they have access to vaccination opportunities. This may include identification of those who are not leaving home, and those without a family or caregiver support system; outreach to determine readiness for and concerns about vaccines; and logistical solutions including transportation and appointment scheduling. Solutions for vaccine distribution should be considered along with other approaches to reach vulnerable older adults to ensure their safety and well-being. In the meantime, until widespread vaccinations are completed, especially for those who hesitate to take the vaccine, encouragement for adherence to safety measures will be necessary considering the ongoing spread of infections. Mask wearing, social distancing, and diligent hygiene remain key components of these recommendations. These critical measures, if taken by older adults as well as their close family members and caregivers, may help many to feel more comfortable in reaching out for support services; leaving their homes for reasons spanning social activities, daily living needs, and shopping; as well as attending medical appointments—thus reducing social isolation risk over time.

### 5.2. Leveraging Personal Determinants of Health

Even prior to COVID-19, perspectives on aging had begun to shift from a focus on negative risk factors to encompass the positive resources within an individual’s control [60]. This approach considers the impact of positive personal resources that could be leveraged to impact health outcomes, highlighting factors such as resilience, purpose in life, optimism, and social connections, as well as physical activity and its role in boosting the other important personal factors [60]. Recent research demonstrates that older adults with a combination of at least one of these attributes tend to have better health outcomes than those who do not; for instance, social support in combination with physical activity through attendance of group fitness settings has demonstrated positive outcomes [60]. These findings suggest that interventions to improve or maintain specific positive attributes could be beneficial, especially during a time when social isolation and the other factors greatly influence one another, with social isolation often holding a bi-directional relationship with resilience, physical activity, and optimism [60]. As previously described, these positive personal resources focusing on resilience, purpose in life, optimism, and social connections have been recently defined as the key personal determinants that older adults can access and utilize later in life to buffer challenges [17]. Boosting the key PDOH during times of crisis could potentially help reduce feelings of social isolation, especially through maintained social connectedness.

## 6. Limitations

Notably, there were a few important limitations of this literature review. First, the search of literature encompassed a short time period, focusing on research published from early 2020 to year-end. Most studies on the impacts of COVID-19 were conducted typically for only a few weeks to about two months; sample sizes were very small and not all focused on older adults. Lastly, reported findings show only short-term impacts, rather than long-term effects of the pandemic, with most research published in a “fast-track” environment following reviews without the typical rigorous evaluation. However, the emerging findings in this review provide a base of knowledge for important future research directions.

## 7. Conclusions

In this timely literature review, we hypothesized that the COVID-19 pandemic has quickly worsened and intensified widespread social isolation among vulnerable older adults, many of whom already faced disconnectedness for various reasons. Furthermore, it was proposed that these impacts of intensifying social isolation could potentially span a lengthy duration, with serious long-term consequences on health outcomes. Notably, our findings confirmed these hypotheses, revealing how the COVID-19 pandemic has complicated social isolation for older adults, highlighting the critical importance of their social connections and need to maintain them even during times of crisis. Notably, serious mounting concerns surround the predicted lasting effects of the pandemic on not only social connectedness but also physical and mental health. As described in this review, a “COVID-19 paradox” has emerged: safety protocols protect older adults but concurrently place them at risk of harm from greater social isolation. While social distancing is critical to prevent spread of the virus, this approach must also be balanced with maintaining social connectedness with approaches adapted for safety and distancing. As this phenomenon continues throughout the ongoing pandemic, fears of a social recession have emerged to describe the downstream ramifications for a population of aging seniors who already face declining physical, mental, and cognitive health.

As this review demonstrates, immediate solutions adapted for safety protocols in the “new normal” of this pandemic are urgently needed, as typical outreach initiatives to combat social isolation are largely on hold. Consequently, this dilemma presents implications for healthcare providers, family caregivers, policy makers, and other stakeholders to quickly develop solutions to reach socially isolated older adults, provide support and resources, and combat feelings of social isolation among them. In fact, solutions adapted for safety may be the trending protocol for future interventions, as many older adults with chronic health conditions or compromised immunity may remain fearful to leave their homes even following widespread vaccination efforts and an eventual end to the pandemic. Thus, greater awareness, cooperation, innovation, and various approaches integrating technological resources are needed to address the consequences of a potential long-term social recession in the coming years.

## Figures and Tables

**Table 1:** Examples of Search Terms and Results Returned.

Search Terms (with Search Filter: 2020)	Results
Older Adults Mental Health Post-COVID US	43
Social Isolation Interventions Older Adults COVID	63
Social Recession Older Adults After COVID	137
Social Recession Older Adults COVID	156
Social Recession Older Adults Pandemic	158
Interventions for Social Recession COVID	247
Definition of Social Recession	289
Social Connections Older Adults COVID	305
Social Isolation Solutions Older Adults COVID	367
Impact of Social Recession COVID	538
Social Recession COVID	620

**Table 2:** Applications to Support Socially Isolated Older Adults.

Category	Application	Cost	Purpose
Social Networking	Skype	Free to download	Individual or group phone/video calls
Social Networking	FaceTime	Free w/Apple products	Phone and video calling
Telehealth	Teladoc	Free to download; Telehealth services depend on insurance	Connect to a doctor by phone; Available 24/7
Telehealth	Doctor on Demand	Free to download; Telehealth services depend on insurance	Face-to-face digital connection with a doctor through video
Prescription Management	GoodRx	Free to download; Membership fee	Finds Rx discounts and coupons
Prescription Management	Medisafe Management	Free to download; Membership fee	Provides medication reminders; Drug interaction warnings
Cognitive Impairment	Be My Eyes	Free to download	Connects visually impaired for help with tasks
Cognitive Impairment	Glide Live Video	Free to download; Subscription fee	On-demand communication with sign language and visuals
Health/ Fitness	Calm	Free to download; Subscription fee	Mindfulness and meditation to reduce stress
Health/ Fitness	Headspace	Free to download; Subscription fee	Relaxation app with meditation techniques
Health/ Fitness	MyFitnessPal	Free	Online diet and exercise tracker

## Data Availability

Not applicable.
